# Supplementary material for: Body Composition Trend in Slovene Adults: A Two-Year Follow-Up
Source: Nutrients. 2024 Nov 28;16(23):4123. doi: 10.3390/nu16234123 (PMC11644650; doi:10.3390/nu16234123)
Supplement: Supplementary file 1 [file nutrients-16-04123-s001.zip › nutrients-3309585-supplementary.pdf]

Table S1. Body mass index and FAT% status of adults and older adults according to BMI and FAT% obesity classification.

Parameter		Initial (n = 432)			Follow-up (n = 432)		
		All	Female	Male	All	Female	Male
According to BMI classification (%)[†]							
Normal (BMI 18.5–24.9 kg/m^2)		59.3	68.3	41.1	54.8	63.0	38.3
Overweight (BMI 25–29.9 kg/m^2)		27.3	19.7	42.6	29.2	23.2	41.1
Obesity 1 class (BMI 30–34.9 kg/m^2)		10.6	9.9	12.1	12.9	10.9	17.0
Obesity 2 class (BMI 35–39.9 kg/m^2)		1.6	1.4	2.1	1.9	2.1	1.4
Obesity 3 class (BMI > 40 kg/m^2)		1.2	0.7	2.1	1.2	0.7	2.1
According to BMI classification (95% CI)[†]							
Normal (BMI 18.5–24.9 kg/m^2)		0.593 (0.544, 0.640)	0.683 (0.625, 0.737)	0.411 (0.329, 0.497)	0.548 (0.499, 0.596)	0.630 (0.571, 0.687)	0.383 (0.302, 0.468)
Overweight (BMI 25–29.9 kg/m^2)		0.273 (0.231, 0.318)	0.197 (0.152, 0.248)	0.425 (0.343, 0.511)	0.292 (0.249, 0.337)	0.232 (0.184, 0.286)	0.411 (0.329, 0.497)
Obesity 1 class (BMI 30–34.9 kg/m^2)		0.106 (0.078, 0.139)	0.099 (0.066, 0.139)	0.121 (0.072, 0.186)	0.129 (0.099, 0.165)	0.109 (0.075, 0.151)	0.170 (0.112, 0.243)
Obesity 2 class (BMI 35–39.9 kg/m^2)		0.016 (0.007, 0.034)	0.014 (0.004, 0.036)	0.021 (0.004, 0.061)	0.119 (0.008, 0.037)	0.021 (0.008, 0.045)	0.014 (0.002, 0.050)
Obesity 3 class (BMI > 40 kg/m^2)		0.012 (0.004, 0.027)	0.007 (0.001, 0.025)	0.425 (0.343, 0.511)	0.292 (0.249, 0.337)	0.007 (0.001, 0.025)	0.021 (0.004, 0.061)
p value			**< 0.001**			**< 0.001**	
According to BMI obesity classification (%)[†]							
Normal		86.6	88.0	83.7	84.0	86.3	79.4
Obese		13.4	12.0	16.3	16.0	13.7	20.6
According to BMI obesity classification (95% CI))[†]							
Normal		0.866 (0.830, 0.897)	0.880 (0.837, 0.916)	0.837 (0.765, 0.894)	0.840 (0.802, 0.874)	0.863 (0.817, 0.900)	0.794 (0.718, 0.858)
Obese		0.134 (0.103, 0.170)	0.120 (0.084, 0.163)	0.163 (0.106, 0.235)	0.160 (0.126, 0.198)	0.137 (0.099, 0.183)	0.206 (0.142, 0.282)
p value			0.216			0.070	
According to FAT% obesity classification (%)[†]							
Female	< 35% (normal)		81.0			78.5	
	> 35% (obese)		19.0			21.5	
Male	< 25% (normal)			78.0			78.0
	> 25% (obese)			22.0			22.0
All	Normal	80.0			78.4		
	Obese	20.0			21.6		
According to FAT% obesity classification (95% CI)[†]							
Female	< 35% (normal)		0.810 (0.759, 0.854)			0.785 (0.733, 0.831)	
	> 35% (obese)		0.190 (0.146, 0.241)			0.215 (0.168, 0.267)	

Parameter		All	Female	Male	All	Female	Male
		Initial (n = 432)			Follow-up (n = 432)		
Male	< 25% (normal)			0.780 (0.703, 0.845)			0.780 (0.703, 0.845)
	> 25% (obese)			0.220 (0.154, 0.297)			0.220 (0.154, 0.297)
All	Normal	0.800 (0.759, 0.837)			0.783 (0.741, 0.822)		
	Obese	0.200 (0.163, 0.241)			0.216 (0.178, 0.259)		
p value			0.471			0.905	

Statistically significant values are shown in bold. †Body mass index (BMI) and FAT% obesity classifications by the WHO. BMI = body mass index, FAT% = total body fat percentage and CI = confidence interval.

Table S2. Trends and variations among adult and older adult groups.

	BM		BMI		FAT%		FFM		PhA	
**Predictors**	**Estimates**	***p* value**	**Estimates**	***p* value**	**Estimates**	***p* value**	**Estimates**	***p* value**	**Estimates**	***p* value**
Intercept	57.16	**< 0.001**	19.91	**< 0.001**	18.26	**< 0.001**	47.11	**< 0.001**	6.36	**< 0.001**
Age	0.18	**< 0.001**	0.09	**< 0.001**	0.20	**< 0.001**	-0.00	0.858	-0.02	**< 0.001**
Sex (M)	18.47	**< 0.001**	2.26	**< 0.001**	-7.76	**< 0.001**	19.87	**< 0.001**	0.82	**< 0.001**
Time	1.11	**< 0.001**	0.36	**< 0.001**	0.36	**< 0.003**	0.58	**< 0.001**	0.01	0.378

Statistically significant values are shown in bold. BM = body mass, BMI = body mass index, FAT = total body fat, FFM = fat-free mass, PhA = whole-body phase angle.

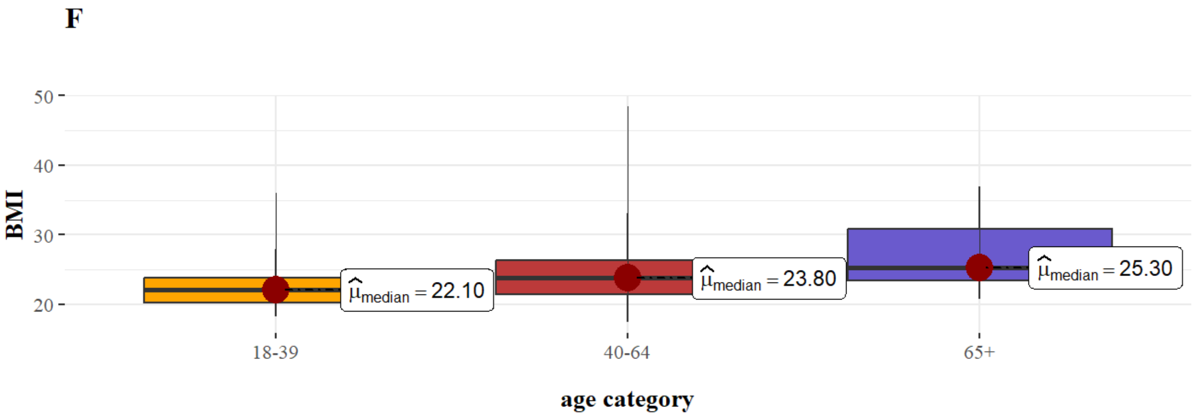

(a) Median BMI of females (initial)

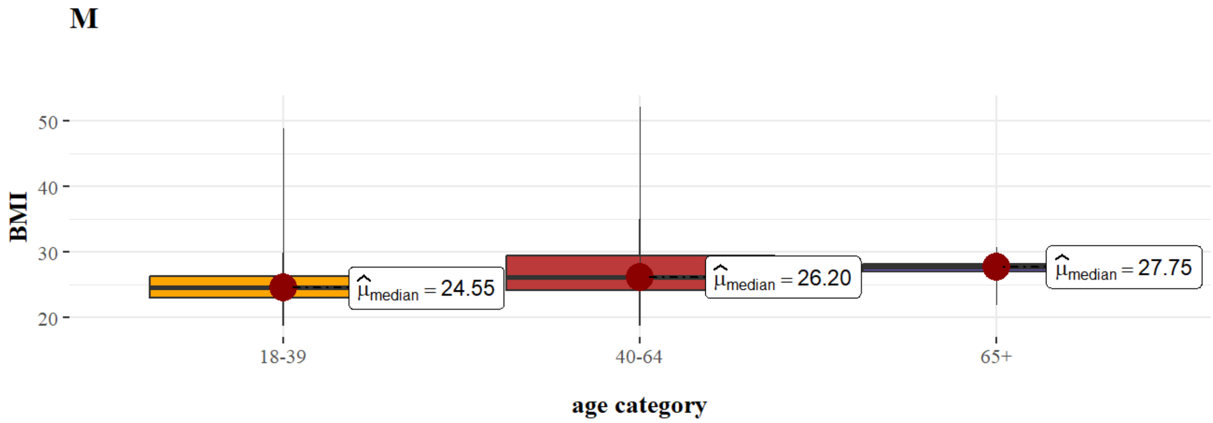

(b) Median BMI of males (initial)

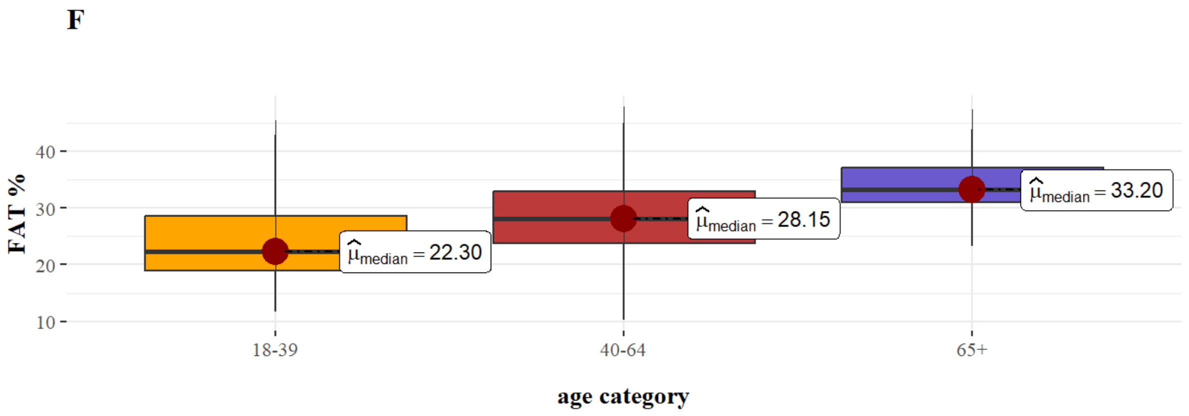

(c) Median FAT% of females (initial)

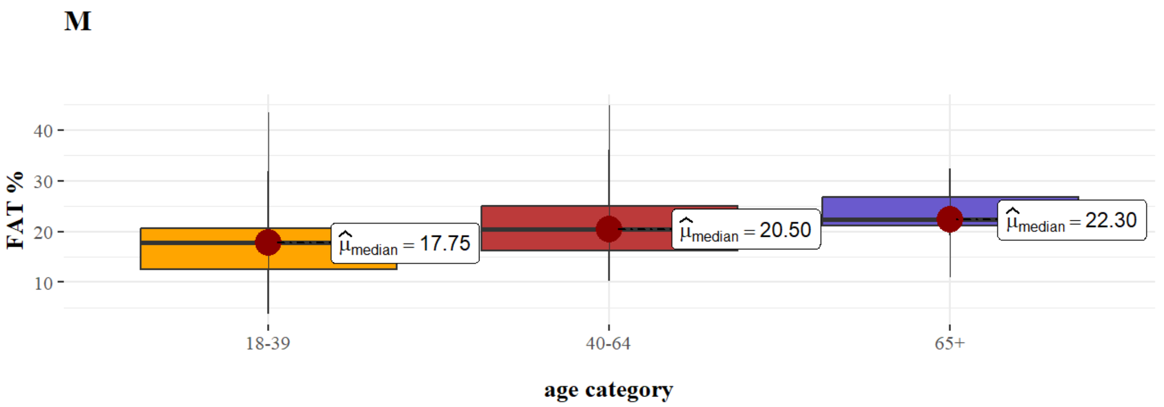

(d) Median FAT% of males (initial)

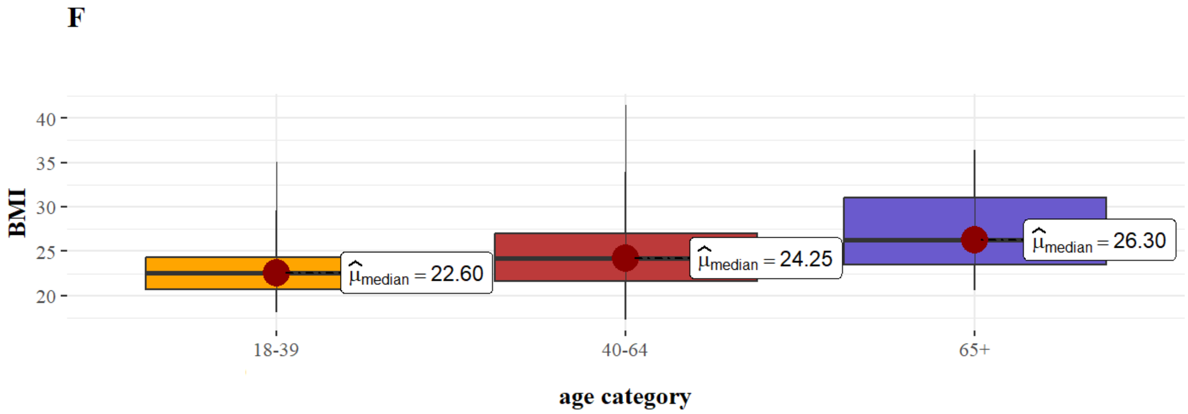

(e) Median BMI of females (FU)

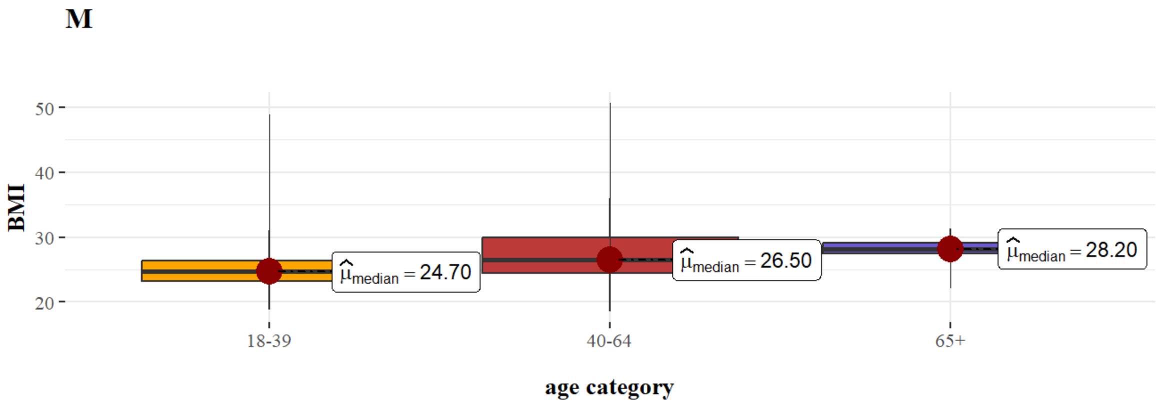

(f) Median BMI of males (FU)

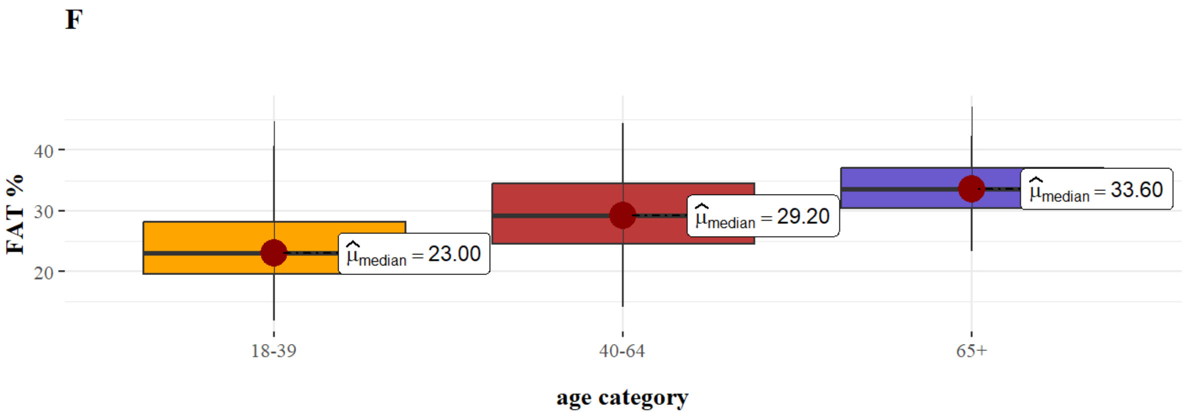

(g) Median FAT% of females (FU)

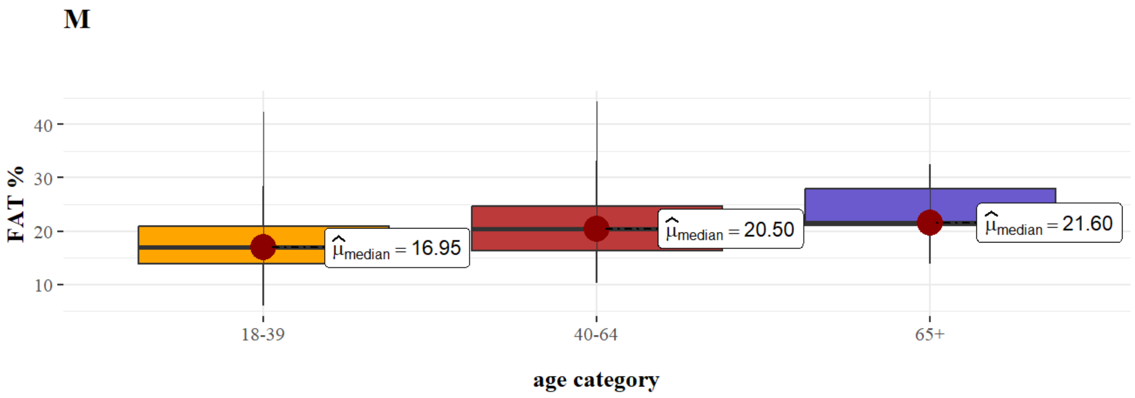

Median FAT% for males (FU)

Figure S1. BMI and FAT% medians according to age categories at the initial and FU.

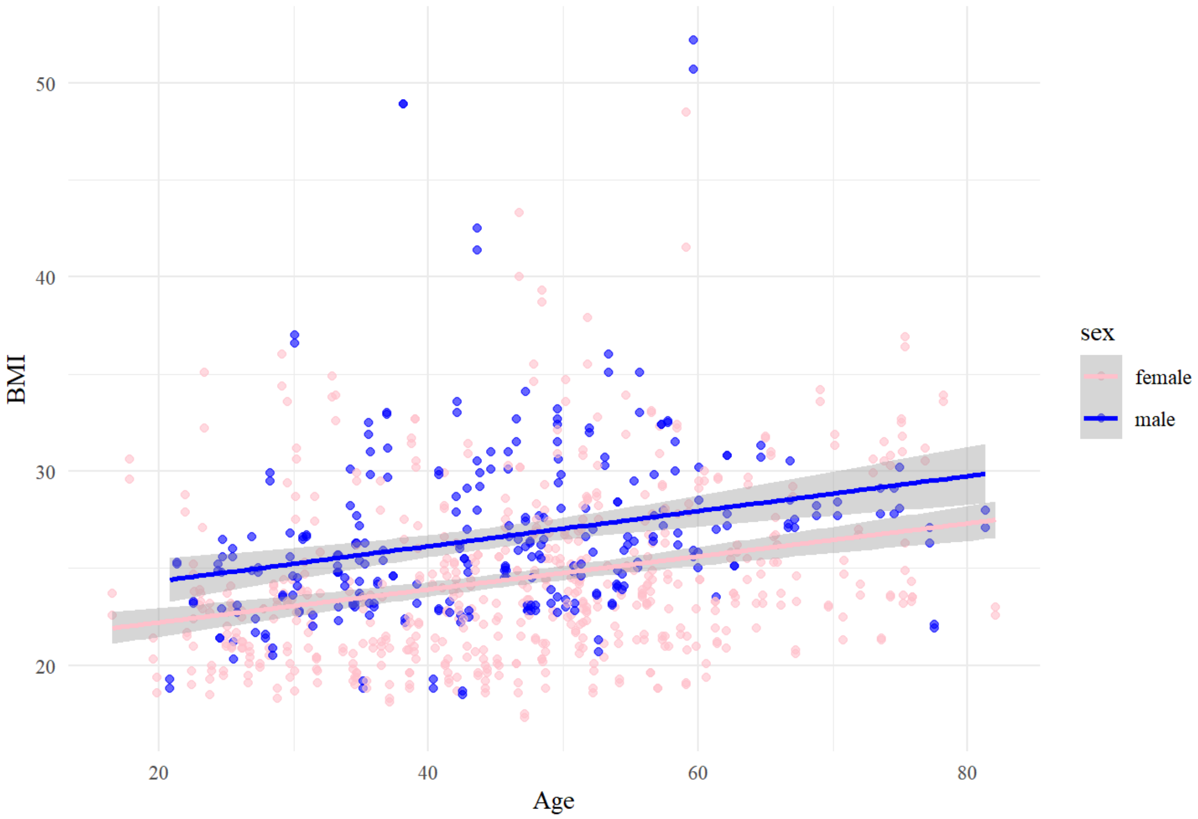

(a)

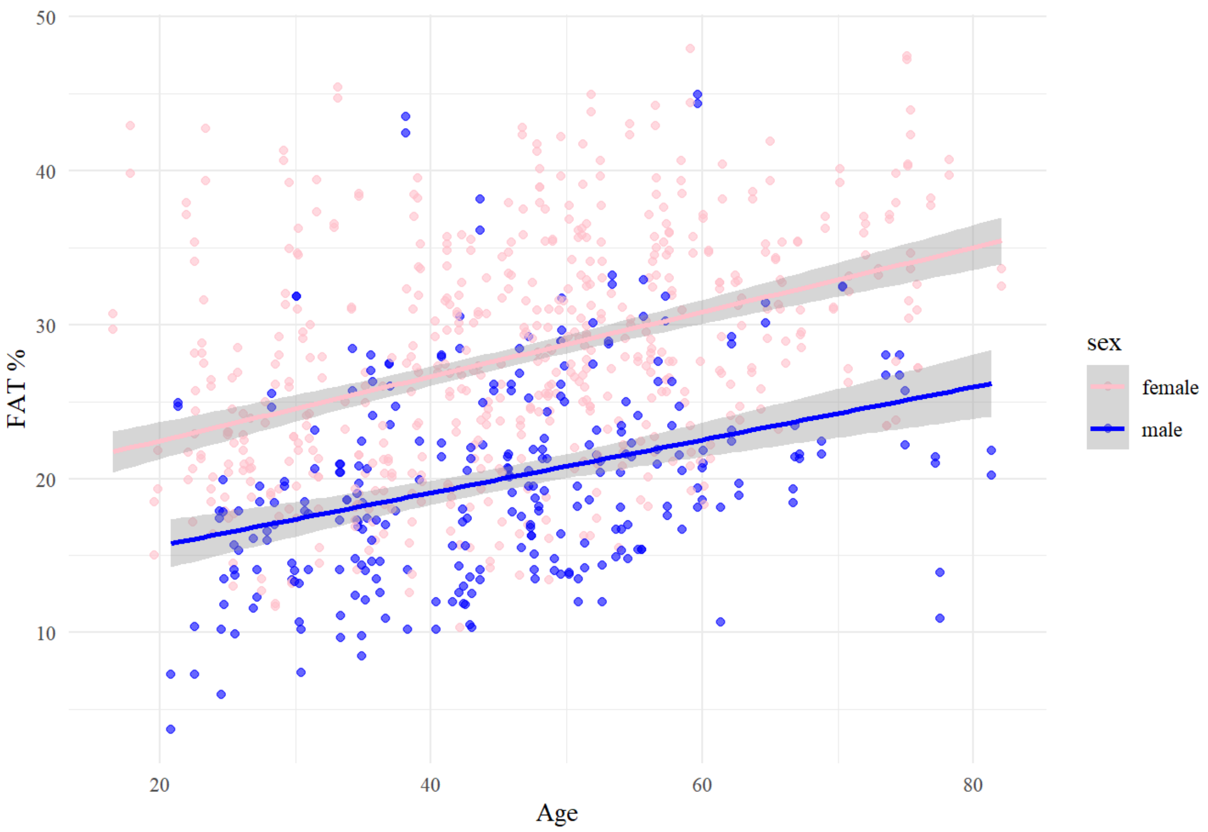
FAT %
Age
sex
female
male
50
40
30
20
10
20
40
60
80

(b)

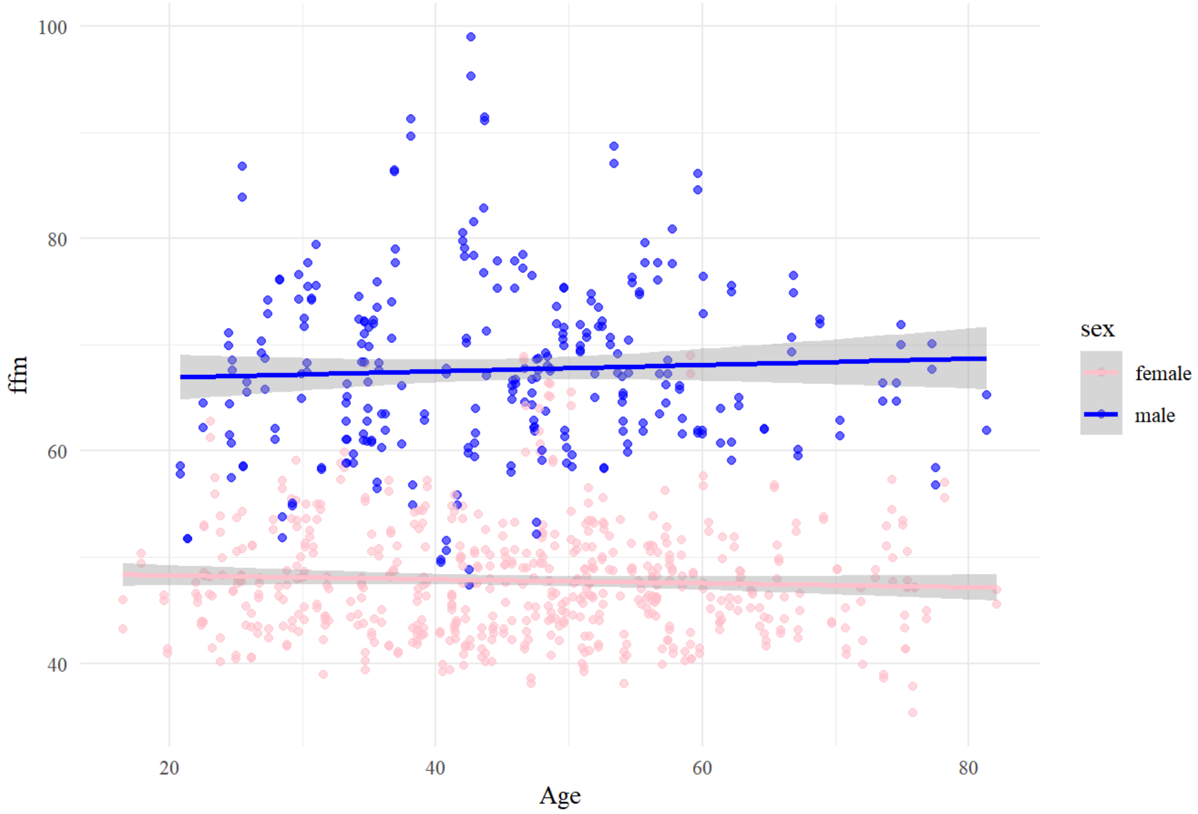
ffm
Age
sex
female
male
100
80
60
40
20
40
60
80

(c)

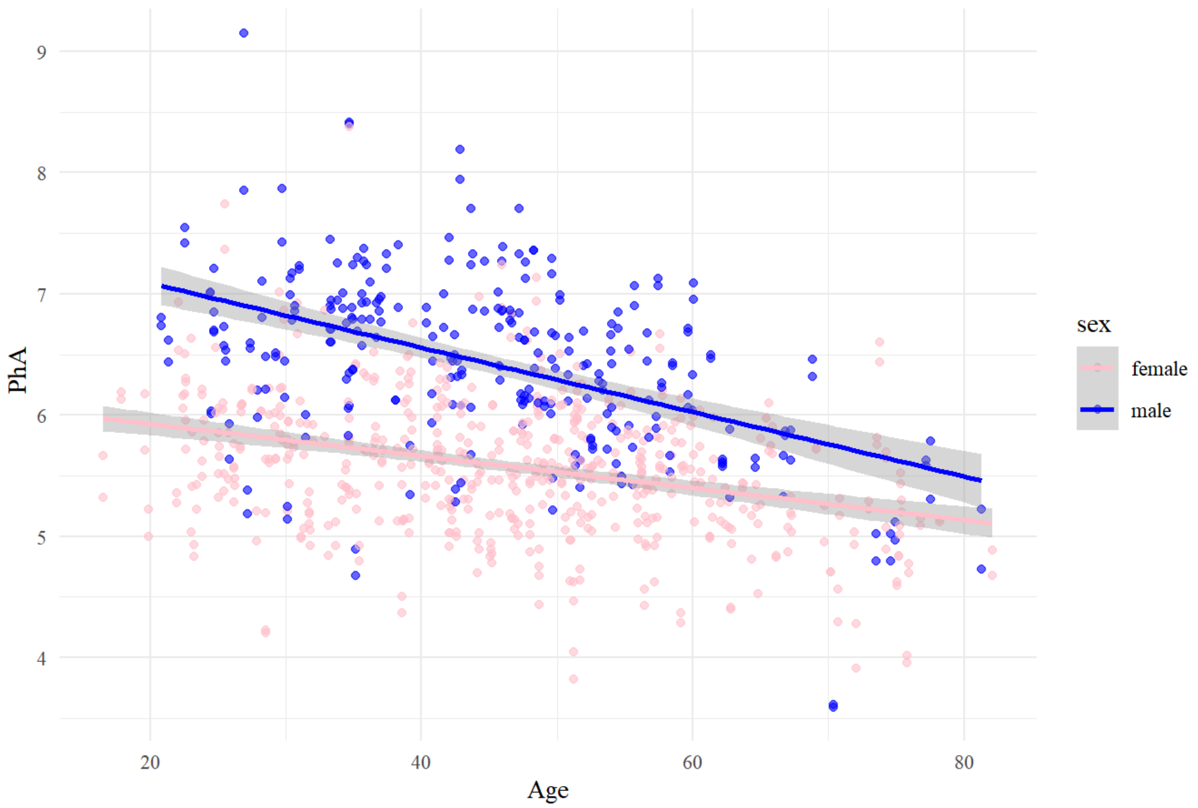

(d) PhA

Figure S2. Distribution of BMI (a), BF% (b), FFM (c), and PhA (d).
